# Two Faces of Protein Kinase Cδ: The Contrasting Roles of PKCδ in Cell Survival and Cell Death

**DOI:** 10.1100/tsw.2010.214

**Published:** 2010-11-16

**Authors:** Alakananda Basu, Deepanwita Pal

**Affiliations:** Department of Molecular Biology and Immunology, University of North Texas Health Science Center and Institute for Cancer Research, Fort Worth, TX, United States

**Keywords:** protein kinase C, PKCδ, apoptosis, cell survival, tumor suppression, tumor promotion, signal transduction, p53, DNA damage–induced apoptosis, receptor-mediated apoptosis

## Abstract

Protein kinase Cδ (PKCδ) is a member of the PKC family that plays a critical role in the regulation of various cellular processes, including cell proliferation, cell death, and tumor promotion. Since the identification that PKCδ is a substrate for caspase-3, there has been overwhelming literature that linked PKCδ with proapoptotic signaling. While PKCδ generally functions as a proapoptotic protein during DNA damage-induced apoptosis, it can act as an antiapoptotic protein during receptor-initiated cell death. PKCδ has also been implicated in tumor suppression as well as survival of several cancers. The function of PKCδ depends on various factors, including its localization, tyrosine phosphorylation, and the presence of other pro- and antiapoptoic signaling molecules. This review discusses the current literature on the contrasting roles of PKCδ in cell survival and cell death.

## INTRODUCTION

Since the discovery that protein kinase C (PKC) is the receptor for tumor-promoting phorbol esters, PKC has been intimately associated with the development and progression of cancer[1]. Although tumor-promoting phorbol esters are potent activators of PKC and mimic the physiologic activator diacylglycerol, persistent treatment with phorbol esters causes degradation or down-regulation of PKC[2,3,4,5]. This raises the question whether activation or down-regulation of PKC is important for tumor promotion. Identification of different PKC isozymes further confounded the role of PKC in cancer. To date, 10 isozymes of PKC have been identified, which are categorized as conventional (α, βI, βII, and γ), novel (δ, ε, η, and θ), and atypical (ζ, λ/ι)[2,6]. These isozymes differ in structure, function, and biochemical properties. Members of PKC isozymes may exhibit overlapping as well as opposite functions[6]. Although it is generally believed that activation of PKCs contributes to cancer, down-regulation rather than activation of PKCδ has been associated with tumor promotion[7]. Thus, PKCδ is believed to function as a tumor suppressor.

PKCδ is not only regulated by cofactors, such as diacylglycerol and phorbol esters, but it is also regulated by phosphorylation. While most PKCs are phosphorylated at the conserved Ser/Thr sites, PKCδ is also phosphorylated at several Tyr residues by various stimuli, including oxidative stress and DNA-damaging agents[6,8,9]. Several tyrosine kinases, including growth factor receptors, Src family tyrosine kinases (e.g., Src, Fyn, Lyn, and Lck), and c-Abl, have been implicated in phosphorylating PKCδ on Tyr residues[8,10,11,12,13]. Depending on the site of phosphorylation and the stimulus, tyrosine phosphorylation of PKCδ can either activate or inhibit PKCδ. In addition, tyrosine phosphorylation of PKCδ can affect its localization, cleavage by caspase-3, as well as its apoptotic function[14].

PKCδ is the first PKC isozyme that was identified as a substrate for caspase-3[15] and there have been numerous studies that linked PKCδ with proapoptotic signaling[3]. It is generally believed that PKCδ and -θ are proapoptotic, whereas PKCα, –β, –ε, –η, –ζ, and -ι are considered antiapoptic[15,16,17,18], despite the fact that PKCε[19] and -ζ[20] are also substrates for caspases. There are two major pathways of cell death. The receptor-mediated or extrinsic pathway is activated upon binding of death ligands to members of the tumor necrosis factor α (TNFα) superfamily. The mitochondrial or intrinsic pathway is activated in response to cellular stress, such as DNA damage. PKCδ has been shown to play a critical role in DNA damage–induced apoptosis[3], although recent studies suggest that it can also regulate receptor-mediated cell death[1]. Numerous studies have used rottlerin, a pharmacological inhibitor of PKCδ, to delineate the role of PKCδ in apoptosis. The specificity of rottlerin has recently been challenged and it is now recognized that rottlerin has many other targets besides PKCδ[21].

It is also important to recognize that PKCδ not only induces apoptosis, but can also function as an antiapoptotic protein and confer resistance to anticancer drugs. Furthermore, PKCδ is required for the survival of several cancers. We reviewed the literature on the involvement of PKCδ in DNA damage–induced apoptosis several years ago[3]. In this review, we discuss the recent literature on the role of PKCδ in apoptosis and try to avoid repetitions of the areas covered in our previous review article[3]. We also highlight the contrasting role of PKCδ as a tumor suppressor and as a prosurvival protein.

## PKCδ AS A TUMOR SUPPRESSOR

Increase in cell proliferation as well as decrease in cell death can lead to the genesis of cancer. Cell cycle checkpoints maintain the genomic integrity of a cell by ensuring the completion of DNA replication and DNA repair before moving on to the next phase[22]. Extensive DNA damage can cause activation of programmed cell death or apoptosis to eliminate damaged cells. The function of PKCδ as a tumor suppressor has been established by the observations that activation of PKCδ inhibits cell cycle progression and down-regulation of PKCδ facilitates tumor promotion[23]. Moreover, PKCδ responds to genotoxic stress by causing cell cycle arrest or by inducing apoptosis[1].

### Tumor Suppression and Cell Cycle Arrest by PKCδ

The PKCδ gene is located on chromosome 3p in a region that is often lost in many cancers, suggesting a role for PKCδ in tumor suppression[24]. It has been shown that ectopic expression of PKCδ decreased anchorage-independent growth of NIH 3T3 cells[25], and reversed transformation of rat fibroblasts[7] and colonic epithelial cells[26] by Src, thus supporting a role for PKCδ in tumor suppression. A decrease in PKCδ expression was also associated with colonic tumors and overexpression of PKCδ suppressed the neoplastic phenotype of colon cancer cells via p53[23]. PKCδ transgenic mice were resistant to 12-O-tetradecanoylphorbol-13-acetate (TPA)–induced tumor promotion[27], but failed to prevent development of squamous cell carcinoma (SCC) in response to UV radiation[28]. A recent report suggests that PKCδ is lost in human SCCs due to transcriptional repression[29]. PKCδ has also been shown to decrease cell migration in breast cancer cells, whereas knockout of the *PKCδ* gene increased cell migration in mouse embryo fibroblasts (MEFs)[30]. A loss of nuclear PKCδ was associated with endometrial tumors[31]. These studies support the function of PKCδ in tumor suppression.

Watanabe et al. first demonstrated that TPA, a tumor-promoting phorbol ester, inhibits the growth of CHO cells overexpressing PKCδ by inducing G2/M arrest[32]. Subsequently, PKCδ was shown to cause both G1/S and G2/M arrest. PKCδ may inhibit cell cycle progression by causing inhibition of cyclin D1 expression, decrease in Cdk1 activity, and increase in levels of cyclin-dependent kinase inhibitors, such as p21 and p27 (reviewed in [23] and [24]). PKCδ-mediated apoptosis was preceded by the initiation of G1 phase cell cycle progression and S phase arrest[33,34]. Recently, it has been demonstrated that the catalytic fragment of PKCδ can phosphorylate Cdk1 at the Tyr15 residue and is important for the maintenance of the G2/M DNA damage checkpoint in response to UV radiation[35]. Since induction of apoptosis is required for the generation of the PKCδ catalytic fragment, this study suggests that G2/M checkpoint activation occurred after apoptosis was initiated.

## Proapopotic Function of PKCδ

The tumor-suppressor protein p53 acts as the guardian of the genome by acting as a master regulator of cellular processes, such as cell cycle arrest, DNA repair, or apoptosis, in response to cellular stress, such as DNA damage[36,37]. Similar to p53-null mice[38], PKCδ-null mice[39,40] developed normally, suggesting that PKCδ is not required for normal cell proliferation. PKCδ-deficient mice were, however, resistant to cell death[32], consistent with its role in apoptosis. Several studies suggest that p53, in fact, acts downstream of PKCδ. Phosphorylation of p53 at Ser46 by PKCδ was shown to be important for p53-mediated apoptosis in response to genotoxic stress[41]. PKCδ also regulates the p53 level by increasing basal transcription of the *p53* gene[42]. It has been reported that upon DNA damage, PKCδ interacts with the death-promoting transcription factor Btf to trigger Btf-mediated *p53* gene transcription and apoptosis[43]. In response to oxidative stress, PKCδ was shown to interact with and activate IKKα in the nucleus[44]. Although IKKα is known to activate NF-κB by phosphorylating IκB at the cytoplasm, PKCδ-mediated activation of IKKα at the nucleus caused phosphorylation of p53 at Ser20, but had no effect on NF-κB activation. However, phosphorylation and level of p53 induced by etoposide or γ-irradiation was not altered in primary parotid cells and parotid glands derived from PKC$\delta^{-/-}$ mice, although the induction of p21 appears to be less in PKC$\delta^{-/-}$ mice compared to wild-type mice[45]. In this study, phosphorylation of p53 at Ser18 (similar to human Ser15) was monitored. Thus, it is not known if etoposide or γ-irradiation induces p53 phosphorylation at Ser45 or Ser20 in PKC$\delta^{-/-}$ mice. PKCδ may also induce apoptosis via mechanisms independent of p53.

It has been reported that PKCδ can trigger DNA damage–induced apoptosis via c-Abl, a nonreceptor tyrosine kinase independent of p53[46]. It is well established that PKCδ can interact with c-Abl at the nucleus to trigger DNA damage–induced apoptosis (reviewed in [8] and [3]). Recently, it has been reported that in response to endoplasmic reticulum (ER) stress, PKCδ translocates to the ER where it interacts with ER-associated c-Abl, and then the PKCδ-Abl complex translocates to the mitochondria to trigger apoptosis[47]. Phosphorylation of PKCδ at Tyr311 by c-Abl was shown to be important for hydrogen peroxide–induced apoptosis[11,48].

Cleavage of PKCδ by caspase-3 separates the catalytic fragment of PKCδ from the autoinhibitory regulatory domain, thereby causing activation of PKCδ in the absence of any cofactors[3]. Several reports have implicated the catalytic fragment of PKCδ in mediating apoptosis (reviewed in [3]). Mutation of the caspase cleavage site of PKCδ prevented UV-induced apoptosis in keratinocytes[49] and proteasome inhibitor–induced apoptosis in dopaminergic neuronal cells[50], but failed to prevent cisplatin-induced apoptosis in human small cell lung cancer cells[51], suggesting that whether or not PKCδ cleavage is needed for the induction of apoptosis depends on the cell type.

Intracellular localization of PKCδ is an important way to reach its targets and thus has significant impact in deciding the ability of PKCδ to induce apoptosis. Constitutively active PKCδ targeted to the cytosol, mitochondria, or nucleus induced apoptosis, whereas PKCδ targeted to the ER protected against tumor necrosis factor–related apoptosis-inducing ligand (TRAIL)– and etoposide-induced apoptosis[52]. PKCδ targeted to the cytosol and mitochondria, but not to the nucleus or ER, underwent proteolytic cleavage, suggesting that proteolytic cleavage of PKCδ was not essential for the induction of apoptosis in the nucleus[52]. This observation was corroborated in etoposide-treated parC5 cells, where nuclear localization of full-length PKCδ was sufficient to induce apoptosis, although cleavage of PKCδ facilitated nuclear retention of PKCδ[53]. Additionally, kinase-negative, full-length PKCδ inhibited apoptosis by preventing nuclear transport of endogenous PKCδ[53]. Once in the nucleus, PKCδ can interact with and/or phosphorylate critical nuclear proteins, such as c-Abl[3,54], p53[41], p73[55], DNA-dependent protein kinase (DNA-PK)[56], lamin[57], Rad9[58], topoisomerase II[59], and heterogeneous nuclear ribonucleoprotein K (hnRNP K)[60] to trigger apoptosis.

Tyrosine phosphorylation of PKCδ has been shown to regulate both nuclear localization of PKCδ and its proteolytic cleavage. Phosphorylation of PKCδ at Tyr64 and Tyr155 in the regulatory domain facilitated nuclear retention of PKCδ[53]. Two tyrosine phosphorylation sites, Tyr311 and Tyr332, near the caspase cleavage site of PKCδ were implicated in regulating proteolytic cleavage of PKCδ by caspase-3. Phosphorylation of PKCδ at Tyr311 by $H_2O_2$ facilitated cleavage of PKCδ and its proapoptotic function during dopaminergic neuronal cell death[61]. In contrast, Tyr332 phosphorylation of PKCδ by Src kinase was necessary for the proteolytic cleavage of PKCδ in response to TRAIL and cisplatin in glioma and HeLa cells, but Tyr311 phosphorylation had no effect[62]. Interestingly, decrease in PKCδ cleavage in the mutant Y332F-expressing cells was not associated with a decrease in caspase-3 activity and an alteration in subcellular distribution or inhibition of PKCδ activity. Overexpression of the Y332F mutant decreased apoptosis induced by the DNA-damaging agent cisplatin, but increased cell death by TRAIL. The authors speculated that phosphorylation of PKCδ at Tyr332 site changes the conformation of PKCδ such that it is more accessible to caspase-3[62].

PKCδ localized at different organelles can influence distinct signaling pathways to regulate apoptosis[52]. PKCδ has been shown to interact with several members of the mitogen-activated protein kinase (MAPK) family, including p38[52], extracellular signal-regulated kinase (ERK)[12,63], and c-jun $NH_2$ terminal kinase (JNK)[45,52]. Cytosolic PKCδ triggered apoptosis by activating p38 MAPK, inhibiting Akt and decreasing the level of X-linked inhibitor of apoptosis protein (XIAP), whereas nuclear PKCδ induced apoptosis via activation of JNK[52]. Phorbol ester–induced apoptosis in prostate cancers involved autocrine secretion of TNF and TRAIL, and activation of p38 MAPK or inhibition of Akt[1]. During etoposide-induced apoptosis, phosphorylation of PKCδ at Tyr64 and Tyr187 caused activation of ERK1/2 by down-regulating MAPK phosphatase-1 (MKP-1)[12]. Bax and Bak are proapoptotic members of the Bcl-2 family that regulate the mitochondrial membrane permeability, thus playing a critical role in apoptosis[64]. It has been reported that upon ionizing radiation treatment, Bax and Bak are activated via the c-Abl-PKCδ-p38 MAPK pathway and trigger the mitochondrial cell death pathway[65,66]. The antiapoptotic Bcl-2 family member Mcl-1 is a direct target of PKCδ[67]. The catalytic fragment of PKCδ was shown to phosphorylate Mcl-1 and target it for degradation, thus facilitating cell death[67]. During the early stages of hypoxic stress, PKCδ was shown to activate autophagy by JNK-mediated phosphorylation of Bcl-2, and dissociation of Bcl-2/beclin 1 complex and prolonged hypoxic stress resulted in the cleavage of PKCδ[68]. The status of other PKC isozymes may also influence the ability of PKCδ to induce apoptosis. Recently, it has been reported that PKCδ triggers apoptosis in cells expressing mutant hyperactive Ras via p73 only when PKCα and -β are present in the cells[69]. Thus, the cellular context plays a major role in deciding the function of PKCδ.

Although PKCδ is a substrate for caspase-3, we have shown that it also acts upstream of caspases[70] and speculate that caspase-3 serves as a substrate for PKCδ[3]. It has now been shown that PKCδ interacts with and phosphorylates caspase-3, and activation of PKCδ precedes caspase-3 phosphorylation during spontaneous and etoposide-induced monocyte apoptosis[71]. Additionally, PKCδ has been shown to be cleaved by human recombinant caspase-2[72], which can function as an apical caspase upstream of caspase-3. The functional significance of caspase-2–mediated cleavage of PKCδ *in vivo* was demonstrated by using the cell-permeable peptide caspase inhibitor VDVAD to prevent PKCδ cleavage and the PKCδ inhibitor rottlerin to prevent doxorubicin-induced apoptosis. We now know that none of the commercially available caspase inhibitors are specific and that rottlerin has many targets. In fact, we have recently demonstrated that rottlerin down-regulates caspase-2 in a PKCδ-independent manner[73]. Thus, it remains to be established whether PKCδ is a substrate for caspase-2 in intact cells and the functional significance of caspase-2–mediated cleavage of PKCδ on apoptosis.

## PROSURVIVAL FUNCTION OF PKCδ

Although PKCδ serves as a critical proapoptotic signal, depending on the cellular context, it can also elicit survival signals. As described below, PKCδ has been shown to promote survival of non–small cell lung cancer (NSCLC), breast cancer, pancreatic cancer, liver cancer, and chronic lymphocytic leukemia cells.

It was reported that rottlerin as well as a kinase-dead mutant of PKCδ, but not wild-type PKCδ, enhanced apoptosis and potentiated chemotherapy-induced apoptosis in NSCLC cells, suggesting that PKCδ promotes cell survival and resistance against chemotherapeutic drugs in NSCLC cells[74]. PKCδ antisense oligonucleotide as well as dominant-negative PKCδ decreased survival of breast cancer MCF-7 and MDA-MB-231 cells[75]. In murine mammary NMuMG cells, PKCδ overexpression increased cell proliferation, anchorage-independent growth, and resistance to apoptotic stimuli by inducing cyclin D1 level and hyperphosphorylation of Rb[76]. PKCδ mRNA level was higher in ER-positive tumors compared to ER-negative tumors, and an increase in PKCδ mRNA was associated with reduced overall patient survival[77].

PKCδ is overexpressed in human ductal pancreatic carcinomas compared with normal counterparts, and ectopic expression of PKCδ increased anchorage-independent growth and resistance to serum starvation and cytotoxic drugs[78]. Interestingly, while the migratory ability of PKCδ-overexpressing PANC1 cells was impaired *in vitro*, these cells were more tumorigenic *in vivo* and developed lung metastasis. PKCδ is also important for the invasion of human liver cancer cells[79]. Claudins are integral to the structure and function of the tight junctions, and an increased expression of claudins is associated with invasiveness of cancer[80,81]. A recent report suggests that claudin 1 imparts invasive capacity to human liver cells via the activation of the c-Abl-PKCδ signaling pathway[79]. PKCδ has also been implicated in the metastasis of melanoma cells. Overexpression of PKCδ increased the metastatic potential of murine BL16 mouse melanoma cells[82]. Integrins are transmembrane proteins that mediate cell-cell and cell-extracellular matrix interactions and maintain the integrity of cellular adhesions, and are involved in cell migration and invasion[83]. A recent study demonstrated that αvβ3 integrin–mediated invasion of melanoma cells is mediated via PKCα and -δ[84].

The pro- and antiapoptotic function of PKCδ not only depends on the cell type, but also on the stimulus. We have shown that rottlerin blocks DNA damage–induced apoptosis, but potentiates receptor-induced apoptosis[85]. Although rottlerin has other targets, these results were corroborated by genetic manipulation of PKCδ[10]. Depletion of PKCδ by siRNA or overexpression of the kinase-dead mutant of PKCδ enhanced TRAIL-induced apoptosis. Interestingly, proteolytic cleavage of PKCδ was necessary for the antiapoptotic effect of PKCδ during TRAIL-induced apoptosis, and phosphorylation of PKCδ at Tyr155 was needed for the translocation of PKCδ to ER and its cleavage by TRAIL. Phosphorylation of PKCδ at Tyr332 was also required for protection against TRAIL-induced apoptosis since mutation of PKCδ at Tyr332 increased TRAIL-induced apoptosis[62].

Overexpression of PKCδ has also been associated with resistance to chemotherapeutic drugs. We have found that the level of PKCδ increased rather than decreased when human cervical cancer

HeLa[86,87] and small cell lung cancer H69 cells[88] acquired resistance to cisplatin. Although proteolytic cleavage of PKCδ is associated with its proapoptotic function, down-regulation of PKCδ rather than the level of the PKCδ catalytic fragment correlated with cisplatin sensitivity in both parental and cisplatin-resistant cells[86,87,89]. Interestingly, the ability of tumor-promoting phorbol esters to down-regulate PKCδ was compromised in cisplatin-resistant HeLa cells[86,87], but knockdown of PKCε or mTOR/rictor could restore activator-induced down-regulation of PKCδ[90], suggesting a cross-talk between PKCδ and other survival pathways. A recent study demonstrated that PKCδ also confers resistance to doxorubicin analogs[91].

PKCδ promotes cell survival via several well-known prosurvival pathways, including NF-κB, Akt, and ERK. PKCδ inhibited apoptosis in colon cancer cells by inducing inhibitor of apoptosis protein-2 (cIAP2)[92] and FLICE-like inhibitory protein (FLIP)[93] via NF-κB. It has been reported that PKCδ promotes survival of pancreatic ductal carcinoma cells by constitutively suppressing autophagy through induction of tissue transglutaminase that has been implicated in drug resistance, metastasis, and poor prognosis[94]. Since overexpression of transglutaminase has been shown to cause activation of NF-κB[95], the authors speculated that PKCδ-mediated suppression of autophagy involves NF-κB.

NF-κB has also been implicated in PKCδ-mediated TNF/TRAIL resistance. Inhibition/knockdown of PKCδ decreased NF-κB and sensitized MCF-7 cells against TRAIL-induced cell death[96]. TNF was shown to induce translocation of PKCδ to the nucleus, where it bound to the NF-κB RelA subunit and induced transactivation of p65/RelA[97]. PKCδ also protected against chemotherapeutic drug-induced apoptosis via NF-κB; inhibition of NF-κB reversed resistance to doxorubicin analogs AD198 and AD288[91].

It has been reported that activation of Akt by oncogenic Ras requires PKCδ activity[98]. An activating mutation of p21 Ras or activation of the phosphatidylinositol 3-kinase (PI3K) increased PKCδ level/activity, leading to the activation of Akt. Activation of both Akt and ERK has been implicated in the PKCδ-mediated increase in anchorage-independent growth and resistance of pancreatic ductal cancer cells to apoptotic stimuli[78]. Increased cell proliferation by overexpression of PKCδ in murine mammary cells was associated with activation of ERK/MAPK[76]. In contrast, suppression of ERK1/2 by PKCδ was associated with the survival of MDA-MB-231 cells[99]. In immortalized and malignant keratinocytes, PKCδ attenuated apoptosis by inducing phosphorylation and proteasomal degradation of the proapoptotic protein Bim via the MEK/MAPK pathway[100]. On the other hand, the tyrosine kinase Syk was shown to promote survival of B-cell chronic lymphocytic leukemia (B-CLL) cells by stabilizing Mcl-1 via PKCδ[101]. Mcl-1 is a substrate for glycogen synthase kinase-3 (GSK3) and phosphorylation of Mcl-1 by GSK3 targets it for proteasome-mediated degradation[102]. Activation of PKCδ by Syk induced phosphorylation and inhibition of GSK3, causing stabilization of Mcl-1 and inhibition of apoptosis. Thus, PKCδ acts in cooperation with other antiapoptotic proteins to promote cell survival.

## CONCLUSION

As discussed in this review article, the function of PKCδ is influenced by several factors, including proteolytic activation of PKCδ, tyrosine phosphorylation, intracellular localization, and the status of other signaling pathways and target proteins. It is difficult to assign a single factor that determines whether PKCδ will promote cell death (Fig. 1) or cell survival (Fig. 2). While cellular context plays a major role in deciding the function of PKCδ, different apoptotic stimuli may elicit opposite effects within the same cell type. Although it is generally believed that proteolytic cleavage of PKCδ is important for apoptosis, the catalytic fragment of PKCδ has been shown to function as both a pro- and antiapoptotic protein depending on the apoptotic stimulus. For example, while the caspase cleavage mutant of PKCδ inhibited DNA damage–induced apoptosis, it enhanced death receptor-mediated apoptosis[10].

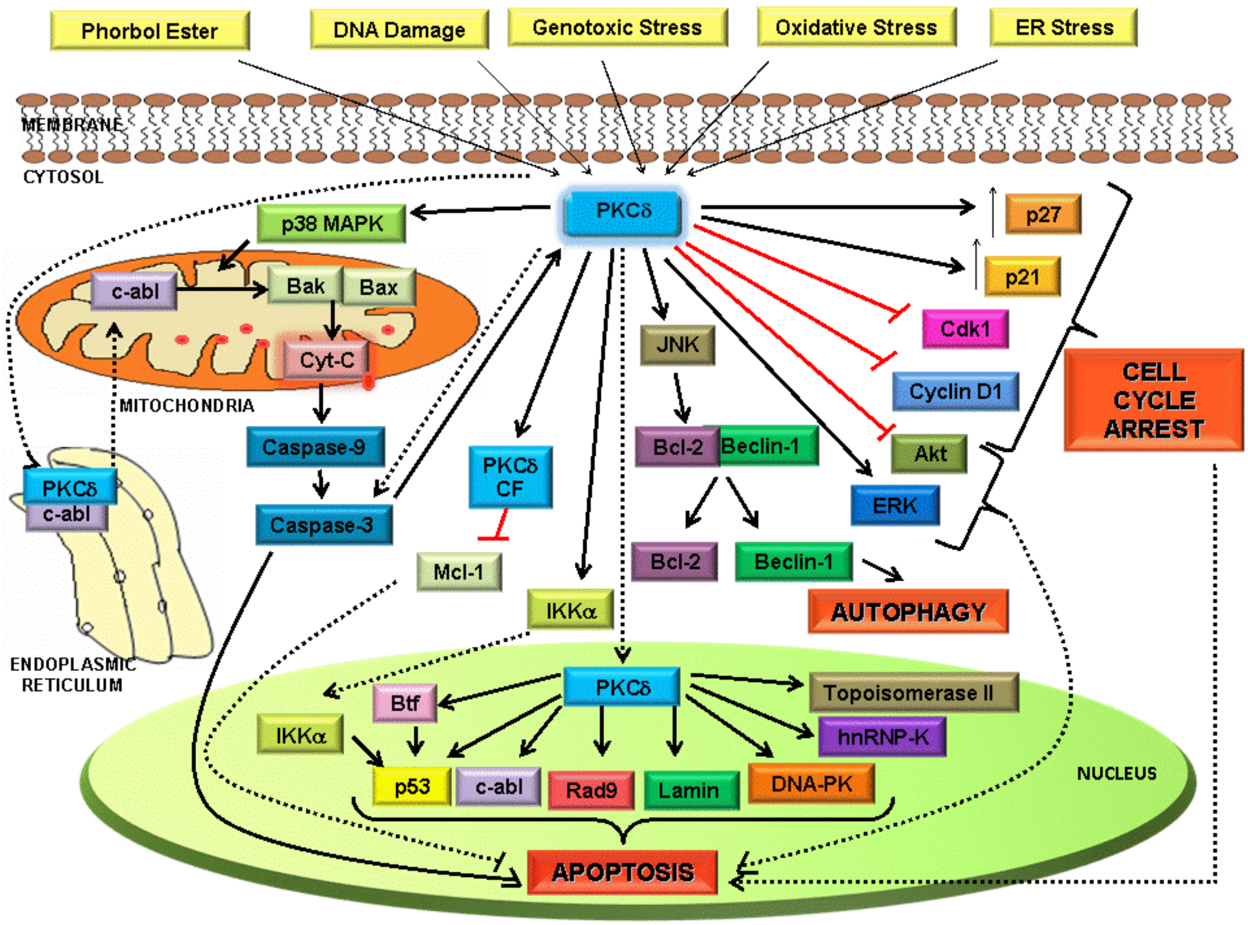

**FIGURE 1.** PKCδ as a tumor suppressor. PKCδ can act as a tumor suppressor in response to tumor-promoting phorbol esters like TPA. Upon DNA damage, it can elicit apoptosis via the p53 pathway or c-Abl. While caspase-3 can proteolytically cleave PKCδ, it can also serve as a target for PKCδ-mediated phosphorylation, thus giving rise to a feedback loop. The catalytic fragment (CF) of PKCδ can enhance apoptosis by inducing phosphorylation and degradation of the antiapoptotic Bcl-2 family protein Mcl-1. PKCδ induces cell cycle arrest by regulating the levels of cyclins, cdks, and cdk inhibitors. It suppresses Akt in response to cellular stress, thus inhibiting survival. It can also regulate autophagy via the JNK pathway through Bcl-2 phosphorylation, causing its dissociation from Beclin-1. The localization of PKCδ to different organelles can stimulate distinct signals, leading to tumor suppression. While PKCδ interacts with c-Abl and regulates members of the Bcl-2 family in the mitochondria, it can interact with several proteins in the nucleus.

Differential intracellular distribution of PKCδ and its cleavage products in response to diverse apoptotic stimuli, such as DNA-damaging agents, TNF/TRAIL, or oxidative stress, may have a significant impact on the pro- and antiapoptotic signaling by PKCδ. In fact, proteolytic cleavage of PKCδ is dispensable for DNA damage–induced apoptosis if PKCδ is retained in the nucleus[52]. Tyrosine phosphorylation of PKCδ can regulate both proteolytic cleavage and intracellular localization of PKCδ. Tyr332 phosphorylation could increase both pro- and antiapoptotic function of PKCδ triggered by DNA damage and death ligands, respectively, by facilitating PKCδ cleavage[62]. Interestingly, TRAIL-induced phosphorylation of PKCδ at Tyr155 was essential for its translocation to the ER and subsequent cleavage in glioma cells[10]. In contrast, Tyr155 phosphorylation facilitated nuclear translocation and retention of PKCδ in response to etoposide, resulting in cell death in salivary epithelial cells[53]. It is not clear why

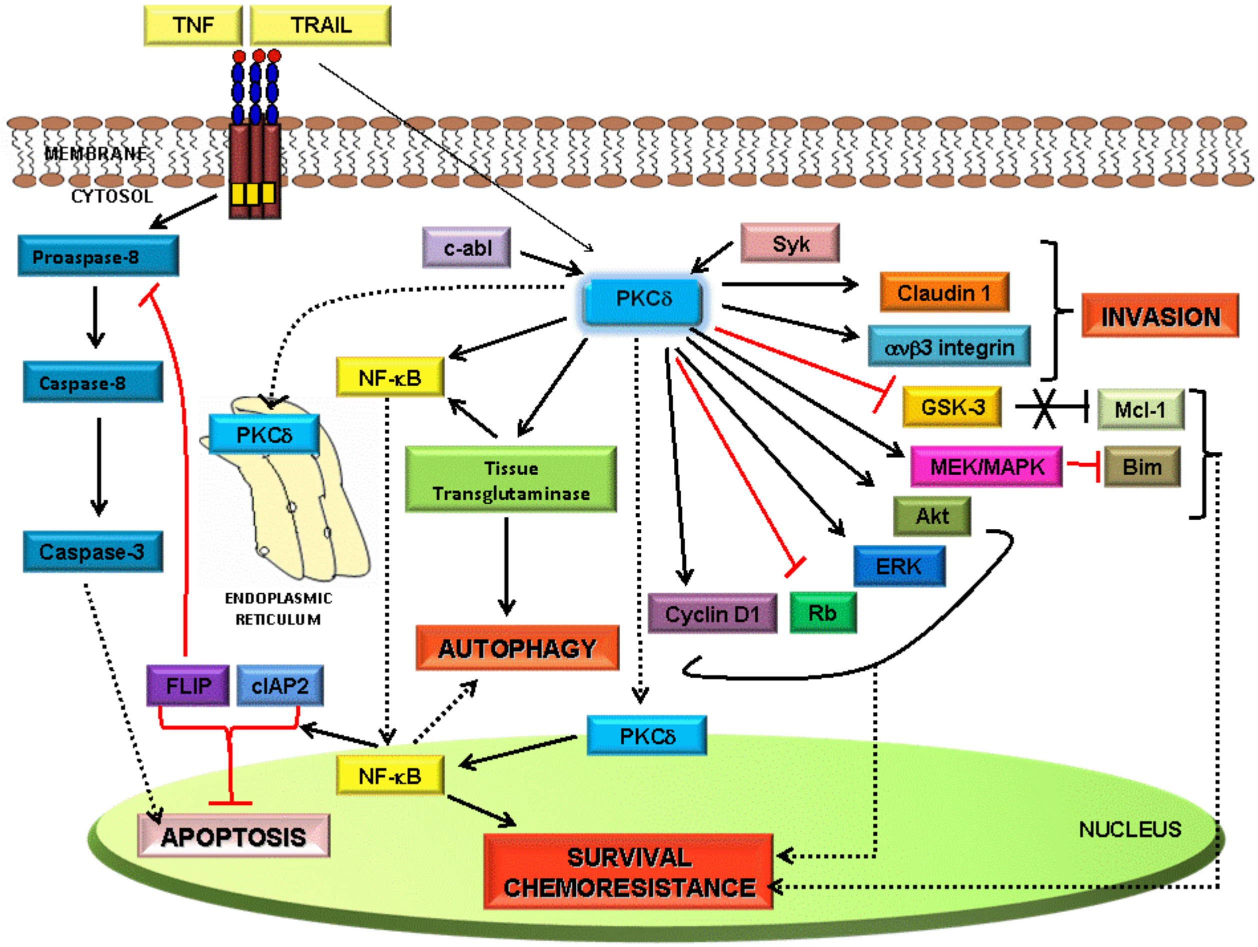

**FIGURE 2.** Prosurvival function of PKCδ. PKCδ can elicit survival signals and promote resistance to chemotherapeutic drugs. It can activate survival pathways like the Akt, NF-κB, and the MEK pathways to induce cell survival. While PKCδ protects from TRAIL-induced apoptosis, it translocates to the nucleus and interacts with NF-κB in response to TNF treatment. PKCδ increases the cyclin D1 level and hyperphosphorylates Rb, thus promoting cellular proliferation. It can promote invasion and metastasis via claudin 1 and αvβ3 integrin. Inhibition of autophagy is also mediated by PKCδ through the induction of tissue transglutaminase and activation of NF-κB. It can inhibit apoptosis via stabilization of antiapoptotic Mcl-1 or degradation of proapoptotic Bim.

phosphorylation at the same site has different fates in response to TRAIL vs. etoposide and if this effect depends on the cell type. These studies are consistent with the notion that translocation of PKCδ to the ER protects against receptor-mediated cell death and nuclear translocation is important for DNA damage–induced apoptosis, although a recent study demonstrated that nuclear translocation of PKCδ is important for the protection against TNF-induced cell death[97]. While studies with ectopic expression of PKCδ and its mutants have provided important insights into the importance of PKCδ tyrosine phosphorylation and its localization in regulating cell survival and cell death, future studies should translate these observations into a physiological context.

Two important transcription factors can mediate the effects of PKCδ on cell survival and cell death. While PKCδ can promote tumor suppression and apoptosis via the tumor-suppressor protein p53 (Fig. 1), it can trigger survival signaling via NF-κB (Fig. 2). Interestingly, several molecules can participate in both pro- and antiapoptotic signaling. For example, while activation of ERK1/2 has been associated with

DNA damage–induced apoptosis[12], it can also contribute to the survival and aggressiveness of cancer[78]. Similarly, while phosphorylation of the antiapoptotic protein Mcl-1 by the PKCδ catalytic fragment led to its degradation and induction of apoptosis[67] (Fig. 1), PKCδ inhibited phosphorylation of Mcl-1 by GSK3, resulting in its stabilization and cell survival[101] (Fig. 2).

It is also difficult to associate a particular tumor type with the prosurvival vs. proapoptotic function of PKCδ. For example, while the tumor-suppressive function of PKCδ in colon cancer is well documented[23], PKCδ was shown to function as an antiapoptotic protein in colon cancer cells by increasing the levels of antiapoptotic proteins c-FLIP and c-IAP2[92,93]. Similarly, it is generally believed that PKCδ is important for the survival of breast cancer cells[75] and an increase in PKCδ correlated with metastatic potential of breast cancer cells[103], yet overexpression of PKCδ inhibited breast cancer cell migration[30]. It is conceivable that PKCδ has distinct functions at different stages of cancer development and progression. Since there is considerable heterogeneity even within a histologic type, it is difficult to correlate the function of PKCδ with a particular tumor type. Although studies with PKCδ transgenic and knockout mice favor the role of PKCδ as a tumor suppressor, it is clear that the apoptotic stimulus as well as the existence of other signaling pathways greatly influence the function of PKCδ in cell survival and cell death.

## ACKNOWLEDGMENT

We sincerely apologize if we inadvertently left out any major contribution in this field. This work was supported by the grant CA071727 (AB) from the National Cancer Institute.
